# Case-matched Comparison of Cardiovascular Outcome in Loeys-Dietz Syndrome versus Marfan Syndrome

**DOI:** 10.3390/jcm8122079

**Published:** 2019-11-29

**Authors:** Kristina Mühlstädt, Julie De Backer, Yskert von Kodolitsch, Kerstin Kutsche, Laura Muiño Mosquera, Jens Brickwedel, Evaldas Girdauskas, Thomas S. Mir, Adrian Mahlmann, Nikolaos Tsilimparis, Axel Staebler, Lauritz Schoof, Heide Seidel, Jürgen Berger, Alexander M. Bernhardt, Stefan Blankenberg, Tilo Kölbel, Christian Detter, Katalin Szöcs, Harald Kaemmerer

**Affiliations:** 1 German Aorta Center Hamburg at Centre of Cardiology and Cardiovascular Surgery, University Medical Center Hamburg-Hospital Eppendorf, 20246 Hamburg, Germany; kristinamuehlstaedt@gmail.com (K.M.); j.brickwedel@uke.de (J.B.); e.girdauskas@uke.de (E.G.); mir@uke.de (T.S.M.); nikolaos.tsilimparis@med.uni-muenchen.de (N.T.); l.schoof@gmx.net (L.S.); al.bernhardt@uke.de (A.M.B.); s.blankenberg@uke.de (S.B.); t.koelbel@uke.de (T.K.); detter@uke.de (C.D.); k.szoecs@uke.de (K.S.); 2 Department of Pediatric Cardiology and Center for Medical Genetics Ghent, Ghent University Hospital, 9000 Ghent, Belgium; julie.debacker@ugent.be (J.D.B.); Laura.MuinoMosquera@uzgent.be (L.M.M.); 3 Department of Cardiology, University Hospital Ghent, 9000 Ghent, Belgium; 4 Institute of Human Genetics, University Medical Center Hamburg-Hospital Eppendorf, 20246 Hamburg, Germany; k.kutsche@uke.de; 5 University Center for Vascular Medicine and Division of Angiology, Department of Internal Medicine III, University Hospital Carl Gustav Carus, Technische Universität Dresden, 01307 Dresden, Germany; Adrian.Mahlmann@uniklinikum-dresden.de; 6 Radiologische Praxis München Grosshadern, 81377 Munich, Germany; info@radiologie-muenchen-harlaching.de; 7 Institute for Human Genetics at Klinikum rechts der Isar, Technical University Munich, 81675 Munich, Germany; Heide.Seidel@mri.tum.de; 8 Department of Medical Biometry and Epidemiology, all at the University Medical Center Hamburg-Hospital Eppendorf, 20246 Hamburg, Germany; berger@uke.de; 9 Department of Congenital Heart Disease and Pediatric Cardiology, Deutsches Herzzentrum München, Technical University München, 80636 Munich, Germany; Kaemmerer@dhm.mhn.de

**Keywords:** aorta, mitral valve, Loeys-Dietz, Marfan syndrome, TGFBR1, TGFBR2, SMAD3

## Abstract

*Background:* Pathogenic variants in *TGFBR1*, *TGFBR2* and *SMAD3* genes cause Loeys-Dietz syndrome, and pathogenic variants in *FBN1* cause Marfan syndrome. Despite their similar phenotypes, both syndromes may have different cardiovascular outcomes. *Methods:* Three expert centers performed a case-matched comparison of cardiovascular outcomes. The Loeys-Dietz group comprised 43 men and 40 women with a mean age of 34 ± 18 years. Twenty-six individuals had pathogenic variants in *TGFBR1*, 40 in *TGFBR2*, and 17 in *SMAD3*. For case-matched comparison we used 83 age and sex-frequency matched individuals with Marfan syndrome. *Results:* In Loeys-Dietz compared to Marfan syndrome, a patent ductus arteriosus (*p* = 0.014) was more prevalent, the craniofacial score was higher (*p* < 0.001), the systemic score lower (*p* < 0.001), and mitral valve prolapse less frequent (*p* = 0.003). Mean survival for Loeys-Dietz and Marfan syndrome was similar (75 ± 3 versus 73 ± 2 years; *p* = 0.811). Cardiovascular outcome was comparable between Loeys-Dietz and Marfan syndrome, including mean freedom from proximal aortic surgery (53 ± 4 versus 48 ± 3 years; *p* = 0.589), distal aortic repair (72 ± 3 versus 67 ± 2 years; *p* = 0.777), mitral valve surgery (75 ± 4 versus 65 ± 3 years; *p* = 0.108), and reintervention (20 ± 3 versus 14 ± 2 years; *p* = 0.112). In Loeys-Dietz syndrome, lower age at initial presentation predicted proximal aortic surgery (HR = 0.748; *p* < 0.001), where receiver operating characteristic analysis identified ≤33.5 years with increased risk. In addition, increased aortic sinus diameters (HR = 6.502; *p* = 0.001), and higher systemic score points at least marginally (HR = 1.175; *p* = 0.065) related to proximal aortic surgery in Loeys-Dietz syndrome. *Conclusions:* Cardiovascular outcome of Loeys-Dietz syndrome was comparable to Marfan syndrome, but the severity of systemic manifestations was a predictor of proximal aortic surgery.

## 1. Introduction

Loeys-Dietz syndrome (LDS) and Marfan syndrome (MFS) are thoracic aortic diseases that exhibit numerous similarities. First, they are heritable with an autosomal-dominant mode. Second, nucleotide variants in specific genes have been identified as causes of the respective disease. Third, both diseases carry a high risk for aneurysm and dissection of the thoracic aorta. Finally, both Loeys-Dietz syndrome and Marfan syndrome may manifest systemic features involving organ systems such as the heart valves, the eyes, the skeletal system, the lungs, the skin and the dura [1]. 

Marfan syndrome was described for the first time in the late 19th century [2]. Diagnostic criteria have been carefully defined, for the first time in 1986 and revised twice subsequently, based on growing knowledge about the clinical and genetic background [3,4,5,6]. The current so-called revised Ghent criteria define aortic root aneurysm and lens luxation as cardinal features of Marfan syndrome [5]. Manifestations in the skeletal, pulmonary and neurologic organ system are accounted for in a systemic score which is also used in the diagnostic algorithm. This latest nosology for the first time also put weight on the identification of pathogenic variants in the Fibrillin1 gene (*FBN1*) which is the causal gene in Marfan syndrome. Loeys-Dietz syndrome was described for the first time in 2005/6 in patients presenting with an apparently more aggressive aortic phenotype and additional clinical features that are uncommon in typical Marfan syndrome including a bifid uvula or cleft palate, hypertelorism and club feet [7,8]. In contrast to Marfan syndrome, lens luxation is not encountered in Loeys-Dietz syndrome and hence is considered a distinguishing diagnostic feature. However, unlike in Marfan syndrome, today there is no international consensus on diagnostic criteria of Loeys-Dietz syndrome. 

Both Loeys-Dietz syndrome and Marfan syndrome are inherited as autosomal dominant traits. The causal gene in Marfan syndrome is the *FBN1* gene with pathogenic variants identified in more than 90% of Marfan phenotypes. The initial description of Loeys-Dietz syndrome reported pathogenic variants in the genes encoding for the transforming growth factor beta receptor 1 (*TFGBR1*) and 2 (*TGFBR2*). Subsequently, at least four other genes involved in the TGFB signaling pathway have been reported in patients presenting with thoracic aneurysm or dissection and systemic features. These genes include the *SMAD2* [9], *SMAD3* [10], *TGFB2* [11] and *TGFB3* [12] genes. In some individuals, additional syndromes have been linked to these genes such as Aneurysm Osteoarthritis syndrome for the *SMAD3* gene [13] and Rienhof syndrome for the *TGFB3* gene [14]. However, in all four genes nucleotide variants were shown to share phenotypic features of Loeys-Dietz syndrome [15].

Survival of Loeys-Dietz syndrome and Marfan syndrome hinges on the cardiovascular manifestations, where repair of the proximal or distal aorta for aneurysms or dissections, surgery for dysfunction of the mitral valve, and surgical reinterventions have the strongest impact on survival [8,16,17,18,19,20,21,22,23,24,25,26]. Early studies describe Loeys-Dietz syndrome as a severe variant of Marfan syndrome [8], but recent investigations also described milder forms of the disease [17,22]. The rationale of our study therefore was to assess if differences in the cardiovascular outcome of Loeys-Dietz syndrome and Marfan syndrome could be confirmed by an age- and sex-matched comparison of both syndromes. Therefore, we performed this observational case-matched comparison of a Loeys-Dietz syndrome group including 83 individuals with a pathogenic variant in the three most common genes identified in Loeys-Dietz syndrome (*TGFBR1*, *TGFBR2*, and *SMAD3)* gene. To minimize selection bias, we established an age- and sex-matched group with Marfan syndrome for comparison. We compared clinical manifestations and outcomes both between the Loeys-Dietz syndrome versus Marfan syndrome groups, and within the Loeys-Dietz syndrome group according to the pathogenic variant in the *TGFBR1*, *TGFBR2*, and *SMAD3* gene.

## 2. Methods

### 2.1. Patients

We recruited 166 consecutive individuals (86 males, mean age 39 ± 18 years; range 4–86 years) in this retrospective, observational case-matched comparison of Loeys-Dietz syndrome and Marfan syndrome from 3 expert centers. Hamburg enrolled 105, Ghent 53, and Munich eight individuals. The Loeys-Dietz syndrome group comprised 43 men and 40 women with a mean age of 38 ± 18 years (range 4–86 years). Genetic testing of the genes *TGFBR1*, *TGFBR2*, or *SMAD3* was performed for clinical suspicion of Loeys-Dietz syndrome in 45 individuals, and by cascade screening in families with Loeys-Dietz syndrome in 38 individuals. The other 83 persons were age and sex-frequency matched individuals fulfilling the Ghent criteria for Marfan syndrome. All subjects gave their informed consent for inclusion before they participated in the study. The study was conducted in accordance with the Declaration of Helsinki, and the protocol was approved by the Ethics Committee of Hamburg, Ghent and Munich (PV4906). We applied STROBE as guideline for study quality [27].

### 2.2. Clinical Manifestations

We analyzed patient charts to assess age at first and final contact to our centers. We assessed the presence of congenital heart anomalies including atrial septal defect, patent ductus arteriosus, or a bicuspid aortic valve based on surgical charts, institutional echocardiographic images or institutional MR angiographies. All three expert centers utilize current echocardiographic and MR technology with current diagnostic imaging criteria for left ventricular and valvular dimensions and functions as specified recently [28,29].

We assessed all clinical features of Loeys-Dietz syndrome and Marfan syndrome in each patient according to established clinical routines as detailed previously both for Loeys-Dietz syndrome [30] and Marfan syndrome [31]. In all centers systemic score points were documented in the charts according to the Ghent nosology [5], and craniofacial severity index points according to Loeys et al [8]. We used the revised Ghent criteria to establish Marfan syndrome. In the absence of a family history of Marfan syndrome, we diagnosed Marfan syndrome with aortic root dilatation combined with ectopia lentis, or a causative *FBN1* mutation, or a systemic score ≥7 points, or with the combination of ectopia lentis with a *FBN1* mutation known to cause aortic dilatation. In the presence of a family history, we diagnosed Marfan syndrome with demonstration of ectopia lentis, or a systemic score ≥7 points, or aortic root dilatation (Z-scores ≥2 standard deviations above the mean with age above 20 years, or Z-scores ≥3 SD above the mean with age below 20 years [5,6].

There are no consensus criteria of Loeys-Dietz syndrome. However, we considered Loeys-Dietz syndrome in all individuals with causative nucleotide change in one of the genes *TGFBR1*, *TGFBR2*, *SMAD3*, *TGFB2*, *TGFB3*, or *SMAD2* [15]. We initiated genetic testing in 45 individuals with a clinical suspicion of Loeys-Dietz syndrome, as defined by the presence of typical cardiovascular manifestations of Loeys-Dietz syndrome or Marfan syndrome, or with systemic features of Marfan syndrome, or with craniofacial features index. The other 38 individuals were identified through cascade screening for a pathogenic variant found in the family. These individuals had milder phenotypes. Eight of these individuals were adolescents from families with severe Loeys-Dietz syndrome, but without formally defined clinical features of Marfan syndrome or Loeys-Dietz syndrome. These eight individuals underwent corroborative genetic testing because they presented with subclinical features such as mild aortic dilatation (Z-score <2), tricuspid valve prolapse, or systemic clinical features not listed in the Ghent systemic score or in the craniofacial severity score (Table 1). We excluded four individuals with pathogenic variants in the *TGFB2*, *TGFB3*, or *SMAD2* gene, because this group was too small for statistical comparison.

We obtained echocardiographic variables according to Rybczynski et al [32], and mitral valve prolapse criteria according to Freed et al [33], where we documented prolapse of the anterior, posterior and both mitral valve leaflets. We assessed aortic root Z-scores according to Gautier et al [34] or Devereux et al in children or adults, respectively. We derived aortic sinus diameters only in subjects, who presented with a native aortic root (Table 2) [35].

### 2.3. Genetic Analyses

DNA was isolated from leukocytes by standard procedures. For Sanger sequencing, the coding region and exon-intron boundaries of the gene of interest were amplified from genomic DNA. Amplicons were directly sequenced using the ABI BigDye Terminator Sequencing Kit (Applied Biosystems, Foster City, CA, USA) and an automated capillary sequencer (e.g. ABI 3500; Applied Biosystems). Sequence electropherograms were analyzed using the Sequence Pilot software SeqPatient (JSI medical systems, Ettenheim, Germany).

For targeted next-generation sequencing (NGS), enrichment of the regions of interest (ROI) was performed with an Illumina Rapid Capture Custom Enrichment kit (Illumina, San Diego, CA, USA) according to the manufacturer’s instructions. Briefly, following fragmentation of genomic DNA, fragmented DNA was amplified and patient-specific (index) adapters were added by PCR. Samples from 12 patients were combined into one single hybridization mix containing target-specific capture probes. The DNA-probe hybrids were then captured with streptavidin beads, and non-targeted DNA fragments as well as unspecific binding were removed by heated washes. Next, the captured DNA library was eluted from the beads, purified and amplified by PCR. The concentration of each library was measured by Qubit fluorometric quantification (Life Technologies, Carlsbad, CA, USA). For generation of clusters and subsequent sequencing of the targeted DNA samples on a flow cell, a sequencing reagent kit from Illumina was used. High-throughput NGS data were generated on an Illumina sequencing platform. ROI sequences were aligned to the human reference genome (hg19) and visualized and evaluated by the use of the Sequence Pilot module SeqNext (JSI Medical Systems, Ettenheim, Germany) [36]. We classified sequence variants as pathogenic or likely pathogenic variants according to the American College of Medical Genetics and Genomics and the Association for Molecular Pathology standards and guidelines [37]. Molecular karyotyping (array comparative genomic hybridization) was carried out on a clinical basis using the 180k Agilent array (Agilent, Santa Clara, CA, USA) with a mean genome wide resolution of 100 kb (AMADID#027676, hg19/GRCh37).

### 2.4. Clinical Events

We screened charts for major cardiovascular events comprising death of any cause, and all events that required hospitalization for treatment of thoracic or abdominal aortic disease or heart valve disorders. Events comprised death, and cardiovascular interventions, including proximal aortic surgery with graft replacement involving the aortic root, distal aortic repair with endovascular or open repair, and mitral valve surgery with reconstruction or replacement of the mitral valve. All aortic procedures were carried out urgently with presence of dissection or rupture. All other aortic procedures were elective, with timing according to the criteria of the current ESC guideline [38].

Proximal aortic surgery comprised aortic root replacement procedures including the aortic-valve-sparing techniques according to David or to Yacoub, and composite valve grafting procedures according to Bentall comprising aortic root replacement with a biological valve or with a mechanical valve as described previously [39] (Table 3). Five of these procedures carried out in Marfan syndrome extended into the aortic arch with usage of a frozen elephant trunk procedure in three as described previously [40] (Table 3). For distal aortic repair we employed open techniques with surgical placement of a prosthesis in the isolated thoracic aortic segment, or in the thoracoabdominal segment, or in the abdominal aorta. We used endovascular techniques only in two individuals with Marfan syndrome, as described previously [41] (Table 3). Surgery of the mitral valve was carried out for severe regurgitation in all instances, with reconstruction of the valve whenever possible [42]. However, replacement with a biological or mechanical valve prosthesis was necessary in some instances (Table 3). We assessed second events including death and reintervention with any second cardiovascular intervention. We performed time to event analyses with baseline date as the date of birth of each individual, and age at event, or age at final contact as recorded in the charts. Only for time to reintervention analysis, we employed the date of initial intervention as baseline.

### 2.5. Statistical Analyses

Unless otherwise specified, we expressed quantitative data as means ± standard deviation and qualitative data as numbers (percentage). We compared characteristics with the Kruskal-Wallis test for continuous data and the generalized Fisher’s exact test for nominal and categorical data. We investigated the influence of Loeys-Dietz syndrome versus Marfan syndrome, genetic cause, systemic score points, and indication for genetic testing on the age at surgery with Kaplan-Meier estimators to calculate the cumulative probability of event, with the Log rank to screen for meaningful differences. For time-to-event analysis, we performed Cox regression analysis. We included variables with *p* < 0.05 in a multivariable model to determine independent predictors with simple rather than stepwise inclusion (Supplementary Tables). We employed receiver operating characteristic (ROC) curve analysis to assess age, systemic score points, and aortic sinus diameters as discriminators of increased versus lower risk for events (Figures S1–S4). In this explorative study we considered *p*-values as descriptive measures with values <0.05 only as indicators of inhomogeneity between groups, where we did not apply methods such as cross validation or bootstrapping. We used IBM-SPSS software (IBM Corp. Released 2013. IBM SPSS Statistics for Windows, Version 22.0. Armonk, NY: IBM Corp) for all statistical tests.

## 3. Results

### 3.1. Clinical Manifestations

In Loeys-Dietz syndrome, when compared to Marfan syndrome, a history of patent ductus arteriosus was more prevalent, and mean craniofacial scores were higher. There was a trend towards higher prevalence of a bicuspid aortic valve in Loeys-Dietz syndrome than in Marfan syndrome (*p* = 0.059). Conversely, mitral valve prolapse and tricuspid valve prolapse were less prevalent, and systemic score points were lower in Loeys-Dietz syndrome than in Marfan syndrome. Left ventricular ejection fraction was higher in Loeys-Dietz syndrome compared to Marfan syndrome (*p* = 0.019), but they did not differ with regard to other echocardiographic criteria (Table 2). Clinical manifestations were comparable between the 3 causative genes in the Loeys-Dietz syndrome group (Table 2). Systemic score points were lower in Loeys-Dietz syndrome detected by cascade screening than in Loeys-Dietz syndrome detected by genetic testing performed for clinical suspicion of Loeys-Dietz syndrome. However, all other clinical manifestations were distributed without incongruence between both groups of indication for genetic testing (Table S1).

### 3.2. Death of Any Cause

**Figure 1:**
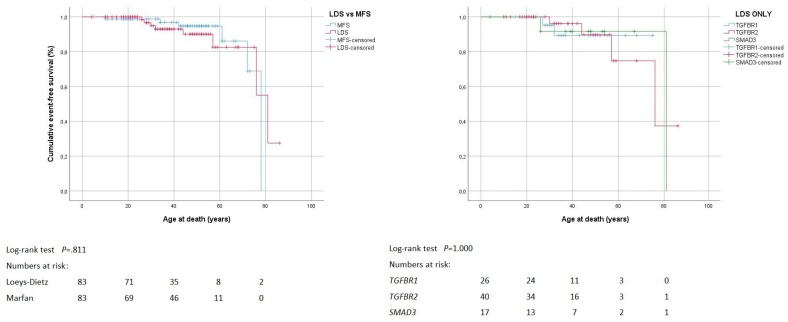
Survival. Kaplan–Meier curve analysis displays the cumulative probability of freedom from death with comparison of both Loeys-Dietz syndrome (LDS) versus Marfan syndrome (MFS; left panel), and LDS within group according to genes *TGFBR1*, *TGFBR2* and *SMAD3* (right panel).

Fourteen deaths occurred with similar frequency and at a similar age in the Loeys-Dietz syndrome and the Marfan syndrome group. The cause of death was unknown in six individuals. The other deaths resulted from aortic complications in five, and from heart failure in three, where heart valve dysfunction was identified as the cause of heart failure in two (Table 3). The mean overall freedom from death in the Loeys-Dietz syndrome group (75 ± 3 years; 95% confidence interval (95% CI) 68–81) was comparable to the Marfan syndrome group (73 ± 2 years, 95% CI 69–78; *p* = 0.811). Within the Loeys-Dietz syndrome group, mean freedom from death was similar with *TGFBR1* (70 ± 3 years, 95% CI 64–77), *TGFBR2* (73 ± 5 years, 95% CI 63–83), and *SMAD3* pathogenic variants (76 ± 6 years, 95% CI 64–89; *p* = 1.000; Figure 1). In Loeys-Dietz syndrome, higher age at initial contact was the only independent predictor of death (hazard ratio (HR) = 0.888; 95%CI 0.823–0.958; *p* = 0.002; Table S2). ROC analysis identified that an age of >31.5 years distinguished higher (78 ± 3 years, 95% CI 72–84) from lower probability of survival (35 ± 1 year, 95% CI 33–37; *p* < 0.001; Figure S1).

### 3.3. Proximal Aortic Surgery

**Figure 2:**
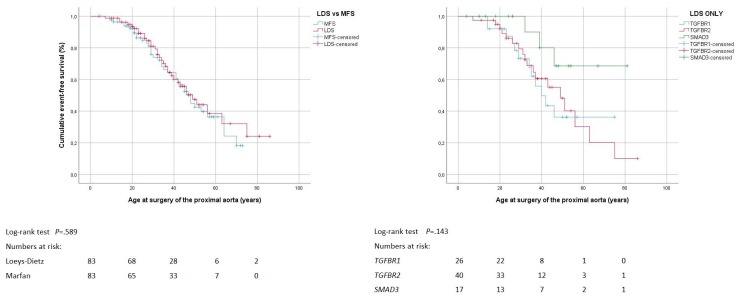
Proximal aortic surgery. Kaplan–Meier curve analysis displays the cumulative probability of freedom from proximal aortic surgery, with comparison of both Loeys-Dietz syndrome (LDS) versus Marfan syndrome (MFS; left panel), and LDS within group according to genes *TGFBR1*, *TGFBR2* and *SMAD3* (right panel).

Seventy individuals had undergone proximal aortic surgery. The frequency, age at surgery, and technique of proximal aortic surgery was comparable, both between the Loeys-Dietz syndrome and the Marfan syndrome group, and between the three causative genes within the Loeys-Dietz syndrome group. Surgery was elective in 48, and urgent in 22 individuals, with similar distribution both between the Loeys-Dietz syndrome and Marfan syndrome group, and between the three causative genes within the Loeys-Dietz syndrome group (Table 3). In seven individuals, comprising two individuals with *TGFBR1* pathogenic variant, and five with Marfan syndrome, proximal aortic surgery was extended into the arch. Of those five individuals with Marfan syndrome, three underwent a frozen elephant trunk procedure. Freedom from proximal aortic surgery was comparable between the Loeys-Dietz syndrome (53 ± 4 years; 95% CI 46–60) and the Marfan syndrome group (48 ± 3 years; 95% CI 43–53; *p* = 0.589; Figure 2). Within the Loeys-Dietz syndrome group, mean freedom from proximal aortic surgery was similar with *TGFBR1* (48 ± 5 years, 95% CI 38–58), *TGFBR2* (49 ± 5 years, 95% CI 39–58), and *SMAD3* pathogenic variants (68 ± 6 years, 95% CI 55–80; *p* = 0.143; Figure 2). 

In Loeys-Dietz syndrome, lower age at initial contact (HR = 0.748; 95% CI 0.658–0.849; *p* < 0.001), and increased aortic sinus diameters were independent predictors of proximal aortic surgery (HR = 4.176; 95% CI 1.721–10.133; *p* = 0.002). Higher systemic score points related marginally to an increased risk of proximal aortic surgery in our multivariate model (HR = 1.175; 95% CI 0.990–1.394; *p* = 0.065), and genetic testing for clinical suspicion versus cascade screening exhibited only univariate association with proximal aortic surgery (Table S3). ROC analysis identified that an age ≤33.5 years at initial presentation distinguished lower (28 ± 1 years, 95% CI 26–31) from a higher probability of freedom from proximal aortic surgery (61 ± 4 years, 95% CI 53–63; *p* < 0.001; Figure S2). ROC analysis also established that a systemic score >2 points separated lower (45 ± 4 years, 95% CI 36–53) from higher probability of freedom from proximal aortic surgery (55 ± 5 years, 95% CI 46–64; *p* = 0.041; Figure S3), whereas the ROC-based cut-off of an aortic sinus diameter > 3.45 cm at initial presentation did not distinguish lower from higher actuarial freedom from proximal aortic surgery (Figure S4). Mean freedom from proximal aortic surgery was lower in Loeys-Dietz syndrome with genetic testing performed for clinical suspicion than in Loeys-Dietz syndrome with cascade screening, whereas freedom from death, from distal aortic repair, and from mitral valve surgery exhibited no inhomogeneity between both indications for genetic testing (Figures S5 and S6).

### 3.4. Distal Aortic Repair

**Figure 3:**
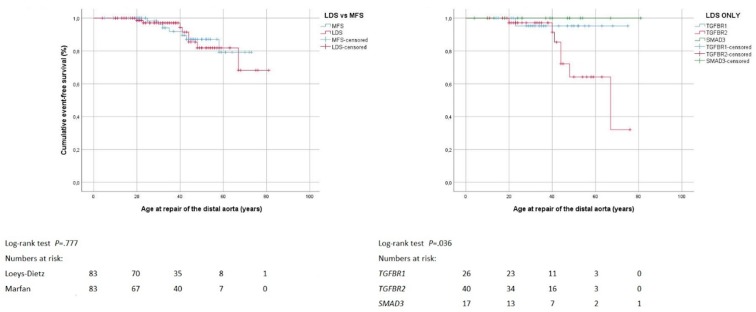
Distal aortic repair. Kaplan–Meier curve analysis displays the cumulative probability of freedom from distal aortic repair, with comparison of both Loeys-Dietz syndrome (LDS) versus Marfan syndrome (MFS; left panel), and LDS within group according to genes *TGFBR1*, *TGFBR2* and *SMAD3* (right panel).

Fifteen individuals underwent distal aortic repair with a comparable frequency and at comparable age in the Loeys-Dietz syndrome and the Marfan syndrome group. Repair was performed urgently for aortic dissection or rupture in two individuals in the Loeys-Dietz syndrome group and in the Marfan syndrome group, respectively. Conversely, repair was performed electively for true or false lumen expansion in six individuals in the Loeys-Dietz syndrome group, and five in the Marfan syndrome group. Distal aortic repair was limited to the thoracic aorta in eight patients, to the abdominal aorta in four patients and involved the thoracoabdominal aorta in three patients. Frequencies were similar in the Loeys-Dietz syndrome and Marfan syndrome group. Two individuals with Marfan syndrome underwent distal aortic repair with endovascular techniques, while all others underwent open surgical repair (Table 3). The Loeys-Dietz syndrome group (72 ± 3 years, 95% CI 66–78) and the Marfan syndrome group showed comparable freedom from distal aortic repair (67 ± 2 years, 95% CI 63–71; *p* = 0.777). Within the Loeys-Dietz syndrome group, freedom from distal aortic repair was lower with pathogenic variants in the *TGFBR2* gene (*p* = 0.036; mean freedom from distal repair was not calculated because all cases were censored; Figure 3). In Loeys-Dietz syndrome, systemic score points (HR = 1.215; 95% CI 1.021–1.445; *p* = 0.028), and the presence of tricuspid valve prolapse showed only univariate association with distal aortic repair (HR = 6.818; 95% CI 1.305–35.610; *p* = 0.023; Table S4).

### 3.5. Mitral Valve Surgery 

**Figure 4:**
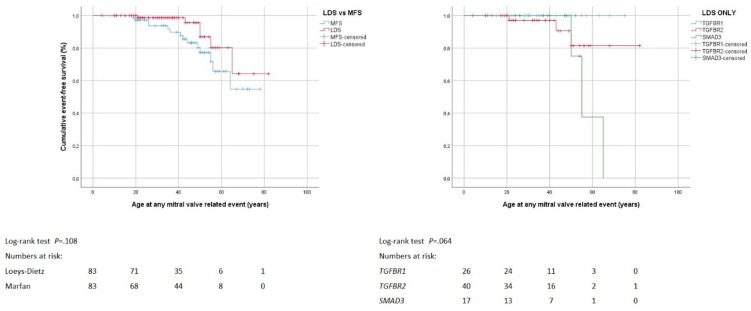
Mitral valve surgery. Kaplan–Meier curve analysis displays the cumulative probability of freedom from mitral valve surgery, with comparison of both Loeys-Dietz syndrome (LDS) versus Marfan syndrome (MFS; left panel), and within LDS group according to genes *TGFBR1*, *TGFBR2* and *SMAD3* (right panel).

Twenty individuals underwent mitral valve surgery with a similar frequency, at comparable age, and with similar techniques in the Loeys-Dietz syndrome and Marfan syndrome group. One individual underwent urgent mitral valve repair for infective endocarditis, whereas all other surgical interventions were elective (Table 3). The Loeys-Dietz syndrome group (75 ± 4 years, 95% CI 67–83) and the Marfan syndrome group showed comparable freedom from mitral valve surgery (65 ± 3 years, 95% CI 59–71; *p* = 0.108). Within the Loeys-Dietz syndrome group, freedom from mitral valve surgery was similar irrespective of the specific gene with pathogenic variant (*p* = 0.064; mean freedom from distal repair was not calculated because all cases were censored; Figure 4). Cox regression analysis did not identify predictors of mitral valve surgery in Loeys-Dietz syndrome (Table S5).

### 3.6. Combined Procedures and Reinterventions

**Figure 5:**
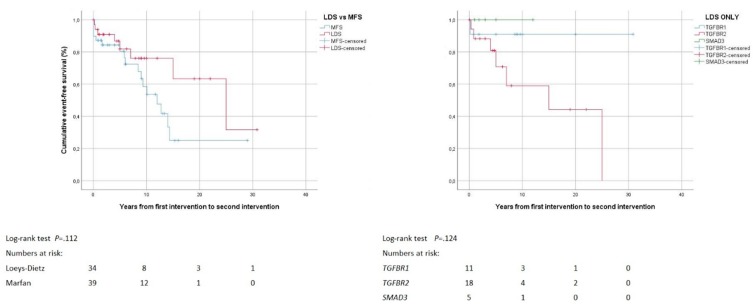
Reinterventions. Kaplan–Meier curve analysis displays the cumulative probability of freedom from reintervention, with comparison of both Loeys-Dietz syndrome (LDS) versus Marfan syndrome (MFS; left panel), and within LDS group according to genes *TGFBR1*, *TGFBR2* and *SMAD3* (right panel).

We performed combined procedures in four individuals with Marfan syndrome that all comprised proximal aortic surgery in conjunction with mitral valve surgery. Thirty-nine individuals with Loeys-Dietz syndrome, and 42 with Marfan syndrome experienced an initial event comprising death or intervention. Among 77 survivors of initial events, 18 (45%) with Loeys-Dietz syndrome, and 11 (30%) with Marfan syndrome experienced a second event. The most frequent reintervention was distal aortic repair in the Loeys-Dietz syndrome group (55%), and mitral valve surgery in the Marfan syndrome group (56%, *p* = 0.006). Distal aortic repair was necessary for false lumen expansion after previous type A dissection in six, for false lumen expansion in chronic type B dissection in three, and for expansion of true thoracic aneurysm in 3 other individuals in the Loeys-Dietz syndrome or in the Marfan syndrome group (*p* = 0.318; Table 3). Mean freedom from reintervention was 20 ± 3 years (95% CI 11–40) with Loeys-Dietz syndrome, and 14 ± 2 years (95%CI 9–18) with Marfan syndrome (*p* = 0.112). Within the Loeys-Dietz syndrome group, mean freedom from reintervention was comparable irrespective the specific gene with pathogenic variant (*p* = 0.124; mean freedom from reintervention was not calculated because all cases were censored; Figure 5).

## 4. Discussion

This multicenter study compared cardiovascular outcome in a large group of Loeys-Dietz syndrome patients to a matched group of individuals with Marfan syndrome. We applied a case-matched design to minimize selection bias in the composition of the Marfan syndrome control group. Loeys-Dietz syndrome was specifically associated with a history of patent ductus arteriosus and increased craniofacial scores. Loeys-Dietz syndrome had lower systemic score points and a lower prevalence of mitral valve prolapse and tricuspid valve prolapse than Marfan syndrome. Our study demonstrated similar risk of death, proximal aortic surgery, distal aortic repair, mitral valve surgery, and cardiovascular reinterventions in Loeys-Dietz syndrome and Marfan syndrome. An increased risk of Loeys-Dietz syndrome for proximal aortic surgery was related to age <33.5 years at initial presentation, to systemic score >2 points, and to a genetic testing performed for clinical suspicion of Loeys-Dietz syndrome rather than for family screening of Loeys-Dietz syndrome.

### 4.1. Clinical Manifestations

As confirmed in the literature, a history of patent ductus arteriosus as well as increased craniofacial scores were common features of Loeys-Dietz syndrome but not of Marfan syndrome. Previous investigations suggested aortic rupture at smaller aortic diameters in Loeys-Dietz syndrome than in Marfan syndrome [5,8]. However, our case-matched study yielded comparable aortic sinus diameters without aortic rupture or dissection, both in Loeys-Dietz syndrome and Marfan syndrome. Our high prevalence of mitral valve prolapse in Loeys-Dietz syndrome confirmed findings in a previous series of *TGFBR1*- and *TGFBR2* pathogenic variants [17,22]. In Marfan syndrome, the prevalence of mitral valve prolapse varied widely across studies, which reflects the use of a wide range of echocardiographic methods and criteria [32]. Therefore, in the current study we uniformly applied standard criteria of mitral valve prolapse [33], and echocardiography was performed exclusively by expert examiners, with joint off-line interpretation of imaging material together with at least one additional board-certified cardiologist. A bicuspid aortic valve was present in 6% of our Loeys-Dietz syndrome group, and in 6% in a surgical series of Loeys-Dietz syndrome in the literature [24]. Our Marfan syndrome group did not include individuals with a bicuspid aortic valve. However, some investigators identified a bicuspid aortic valve in up to 5% of individuals with Marfan syndrome [43,44]. Our study observed preserved systolic left ventricular function and normal left ventricular dimensions in a large group of individuals with Loeys-Dietz syndrome, but myocardial dysfunction and diffuse myocardial fibrosis is known to occur in Loeys-Dietz syndrome [45].

### 4.2. Death of Any Cause

In our study mean freedom from death was strikingly similar in Loeys-Dietz syndrome (75 ± 3 years) and Marfan syndrome (73 ± 2 years). Recent literature corroborated similar survival in individuals with *TGFBR2* and *FBN1* pathogenic variants [17], and showed no difference between *TGFBR1* und *TGFBR2* pathogenic variants [22]. Earlier studies of Loeys-Dietz syndrome were likely to include younger individuals with malignant phenotypes who had not undergone structured management revealing significant attrition due to systemic complications during the follow-up [8]. In our study, death resulted from aortic complications or from heart failure, but the cause of death was unknown in 50% of Loeys-Dietz syndrome. The literature confirmed aortic disease as a leading cause of death in Loeys-Dietz syndrome [22,25], but it also underpinned that the cause of death was unknown in a large number of cases [23,24]. In our study, the only predictor of death was increased age, where we established >31.5 years at initial presentation as threshold of increased risk. Individuals with delayed initial presentation had limited prospect of survival because they had already experienced complications such as acute aortic dissection.

### 4.3. Proximal Aortic Surgery

In our study the frequency and mean age at proximal aortic surgery was similar in both syndromes. Recent studies corroborate a similar frequency of aortic surgery in Loeys-Dietz syndrome and Marfan syndrome, which is carried out at a similar age in both syndromes [17,24,25]. In both groups, Loeys-Dietz syndrome and Marfan syndrome, 30% of surgeries of the proximal aorta were carried out urgently for acute dissection and rupture. The prevalence of type A aortic dissection in Loeys-Dietz syndrome was as high as 64% in some clinics [21], but most high-volume centers report frequencies of type A dissection between 15% and 20% [22,23]. In our centers, in all individuals with acute dissection or rupture of the proximal aorta the diagnosis of Loeys-Dietz syndrome was unknown prior to urgent surgery. This finding reflects the problem of time delay in the diagnosis of rare diseases such as Loeys-Dietz syndrome or Marfan syndrome [6]. Finally, in our series, 17% of *TGFBR1* pathogenic variants required extension of proximal aortic surgery into the aortic arch, where others reported aortic arch replacement in 11%, 18% and 45% of Loeys-Dietz syndrome patients [21,24,25]. A surgical series of Loeys-Dietz syndrome confirmed high rates of proximal aortic surgery, where 18% with *TGFBR1*, 48% with *TGFBR2*, and 27% with *SMAD3* pathogenic variants underwent surgery [25].

Besides enlarged aortic sinus diameters, our study identified (i) higher systemic score points, (ii) lower age at initial presentation, and (iii) genetic testing performed for clinical suspicion of Loeys-Dietz syndrome as potential risk factors of proximal aortic surgery. These predictors were likely to reflect more severe phenotypes of Loeys-Dietz syndrome, where (i) higher systemic score points indicated severe systemic manifestations of Loeys-Dietz syndrome. Accordingly, presence of more severe phenotypes may have prompted both (ii) lower age at presentation, and (iii) clinical suspicion of Loeys-Dietz syndrome as indication for genetic testing. 

### 4.4. Distal Aortic Repair

In our study, 10% of Loeys-Dietz syndrome and 8% of Marfan syndrome required distal aortic repair. The literature reports surgery of the descending in 4% of *TGFBR2* and in 2% of *FBN1* pathogenic variants carriers, which is similar to our findings [17]. In our study, distal aortic repair was carried out urgently for acute aortic dissection or rupture in 25% of Loeys-Dietz syndrome, and 29% of Marfan syndrome. The literature reported acute dissection of the descending aorta in 3% [23] and 13% [25] of Loeys-Dietz syndrome. In our study, distal aortic repair involved the thoracic or the thoracoabdominal part of the descending aorta in 1% with Loeys-Dietz syndrome and 2% with Marfan syndrome, where others reported thoracoabdominal repair in 4% with Loeys-Dietz syndrome [24]. All of our Loeys-Dietz syndrome patients underwent open surgical repair, but placement of stent grafts was reported in these patients [46]. Our study demonstrated freedom from distal aortic repair until 72 ± 3 and 67 ± 2 years of age in Loeys-Dietz syndrome and Marfan syndrome, respectively.

### 4.5. Mitral Valve Surgery

In our study, the prevalence of mitral valve prolapse was lower in Loeys-Dietz syndrome than in Marfan syndrome, but the frequency of mitral valve surgery was similar in both groups. Our study identified a somewhat higher frequency of mitral valve surgery with *SMAD3* (18%) compared to *TGFBR2* (8%), and *TGFBR1* (0%) pathogenic variants. Similarly, a Norwegian series of Loeys-Dietz syndrome found only individuals with *SMAD3* pathogenic variants to require mitral valve surgery, with a high frequency of 5 out of 11 individuals [23].

### 4.6. Combined Procedures and Reinterventions

In our study, combined procedures were performed in 10% of Marfan syndrome, but not in Loeys-Dietz syndrome. However, a surgical series of 33 individuals with Loeys-Dietz syndrome and aortic surgery reported a broad spectrum of concomitant procedures including mitral valve surgery and coronary artery bypass grafting in two individuals, respectively, and septal myectomy and atrial septal aneurysm repair in one patient, respectively [25]. Our study showed that the frequency of reintervention was slightly lower and freedom from reintervention tended to be longer with Loeys-Dietz syndrome compared to Marfan syndrome. Previous studies emphasized a high frequency of reintervention in Loeys-Dietz syndrome [21,24,25], but Patel et al confirmed considerable (47.8%) freedom from subsequent operations after cardiovascular surgery in Loeys-Dietz syndrome at 10 years after initial surgery in 79 individuals with Loeys-Dietz syndrome [24]. In our study, reintervention was necessary at the distal aorta for false lumen expansion after dissection rather than for true aneurysm formation.

### 4.7. Study Limitations

The sample size of our study was limited, and larger multicenter studies are needed for better generalization of study results. However, all three participating centers recruited their patients from the populations of their respective metropole area or country, who represented a relatively unbiased cohort of consecutive individuals with Loeys-Dietz syndrome and Marfan syndrome. The outcome variables death of any cause, distal aortic repair, and mitral valve surgery exhibited less than 10 events, and therefore hazard ratios of Cox regression analysis needed to be considered with caution. However, we double checked associations of variables with events also with Kaplan-Meier curve analysis, where we employed ROC-based thresholds for continuous variables. Unlike with Marfan syndrome, there is no international consensus guideline on diagnostic criteria of Loeys-Dietz syndrome, which may account for variability of Loeys-Dietz syndrome phenotypes with heterogeneous cardiovascular outcomes across different cohorts of Loeys-Dietz syndrome reported in the literature. Finally, we did not consider genes encoding TGF-beta ligands *TGFB2* and *TGFB3*, or *SMAD2*, although these were described recently to contribute to Loeys-Dietz syndrome [15]. The main reason was that only four individuals in our three centers exhibited nucleotide variants in these genes which was not enough for statistical analysis in this study.

## 5. Conclusions

Cardiovascular outcome of Loeys-Dietz syndrome was comparable to Marfan syndrome, but the severity of systemic manifestations is a predictor of proximal aortic surgery. However, large multicenter studies may be necessary to further elucidate the impact of aortic and systemic features on cardiovascular outcome in Loeys-Dietz Syndrome.

## Figures and Tables

**Table 1:** Indication for corroborative genetic testing in 83 individuals of the Loeys-Dietz group.

Variable	Indication for Corroborative Genetic Testing		
	Clinical Suspicion	Cascade Screening	*p*
Total number of individuals	45	38	
Cardiovascular features			<0.001
No cardiovascular features	5 (11%)	16 (42%)	
Isolated aortic features	6 (13%)	1 (3%)	
Isolated mitral valve features	3 (7%)	6 (16%)	
Isolated malformation (ASD, BAV, or PDA, or combinations)	3 (7%)	3 (8%)	
Stroke	0	2 (5%)	
Combination of these cardiovascular features	28 (62%)	10 (26%)	
Systemic features			0.128
No formal systemic features	5 (11%)	11 (29%)	
Isolated systemic features (according to Ghent score)	25 (56%)	18 (47%)	
Isolated craniofacial features (according to Loeys et at.)	15 (33%)	9 (24%)	
Cardiovascular plus systemic features			<0.001
No formally defined clinical features ^1^	0	8 (21%)	
Isolated systemic features (according to Ghent score)	4 (9%)	0	
Isolated craniofacial features (according to Loeys et at)	5 (11%)	8 (21%)	
Cardiovascular and systemic features	36 (80%)	22 (58%)	

ASD identifies atrial septal defect; BAV, bicuspid aortic valve; and PDA, history of patent ductus arteriosus. ^1^ Individuals with no formally defined clinical features of Marfan syndrome or Loeys-Dietz syndrome were adolescents or younger adults who came from families with severe Loeys-Dietz syndrome. They underwent corroborative genetic testing because they presented with subclinical features such as mild aortic dilatation (<2 Z-scores), tricuspid valve prolapse, or systemic clinical features not listed in the Ghent systemic score or in the craniofacial severity score.

**Table 2:** Clinical manifestations at initial presentation.

Variable	Syndrome Group			Loeys-Dietz Group by Causative Genes			
	Loeys-Dietz	Marfan	*p*	*TGFBR1*	*TGFBR2*	*SMAD3*	*p*
Total number of individuals	83	83		26	40	17	
Age at initial contact (years)	34 ± 18	34 ± 18	0.873	34 ± 17	34 ± 18	35 ± 22	0.983
Age at final contact (years)	38 ± 18	40 ± 18	0.523	39 ± 17	38 ± 18	37 ± 21	0.898
Male sex	43 (52%)	43 (52%)	1.000	12 (46%)	20 (50%)	11 (65%)	0.446
Previous ischemic neurologic event	6/80 (8%)	4 (5%)	0.538	2 (8%)	3/39 (8%)	1/15 (7%)	1.000
Atrial septal defect	4 (5%)	1/82 (1%)	0.367	1 (4%)	3 (8%)	0	0.812
History of patent ductus arteriosus	7 (8%)	0	0.014	1 (4%)	6 (15%)	0	0.178
Bicuspid aortic valve	5 (6%)	0	0.059	1 (4%)	2 (5%)	2 (12%)	0.591
Systemic score (points)	3.5 ± 3.5	6.6 ± 3.2	<0.001	3.4 ± 3.8	4.2 ± 3.7	2.1 ± 2.1	0.176
Craniofacial severity index (points)	1.3 ± 1.8	0	<0.001	0.7 ± 1.4	1.6 ± 2	1.3 ± 1.7	0.227
LV ejection fraction (%)	62 ± 11	57 ± 12	0.019	62 ± 10	60 ± 12	68 ± 8	0.103
Indexed LVESD (mm/m^2^)	19 ± 6	19 ± 6	0.392	19 ± 6	19 ± 6	16 ± 3	0.345
Indexed LVEDD (mm/m^2^)	29 ± 8	29 ± 8	0.867	29 ± 7	30 ± 10	27 ± 3	0.754
Indexed left atrial diameter (mm/m^2^)	19 ± 5	19 ± 6	0.663	18 ± 5	20 ± 6	19 ± 5	0.493
Aortic sinus dimensions at initial presentation							
Diameter (cm) ^1^	3.6 ± 0.8	3.6 ± 0.8	0.701	3.3 ± 0.5	3.8 ± 0.7	3.4±1.3	0.102
Z-score ^1^	2.2 ± 3.2	2.4 ± 2.9	0.761	1.8 ± 1.9	2.2 ± 3.6	2.5±3.6	0.675
Aortic sinus dimensions at aortic surgery							
Diameter (cm)	4.8 ± 0.9	5.1 ± 0.9	0.208	4.7 ± 0.3	4.8 ± 0.8	5.0	0.688
Z-score	5.0 ± 3.2	6.7 ± 3.1	0.091	5.3 ± 1.5	4.8 ± 3.1		0.751
Moderate degree of MVR at baseline	7/78 (9%)	11/82 (13%)	0.457	2/25 (8%)	4/39 (10%)	1/14 (7%)	1.000
MV prolapse	28 (34%)	48 (58%)	0.003	5 (19%)	17 (43%)	6 (35%)	0.152
MV leaflet prolapse location (N)	19	38	0.063	5	11	3	0.162
Isolated anterior	10 (53%)	11 (29%)		4 (80%)	6 (55%)	0	
Isolated posterior	2 (11%)	1 (3%)		0	2 (18%)	0	
Combined anterior and posterior	7 (37%)	26 (68%)		1 (20%)	3 (27%)	3 (100%)	
Tricuspid valve prolapse	5/79 (6%)	33/82 (40%)	<0.001	1/25 (4%)	4 (10%)	0/14	0.570

LV identifies left ventricle; LVEDD, LV end-diastolic diameter; LVESD, LV end-systolic diameter; MV, mitral valve; MVR, MV regurgitation; and N, number of individuals. If less than total, we present the number of individuals with available information behind a slash. ^1^ Aortic root diameters were obtained at initial presentation to our centers only in those 126 individuals who presented with native aortic roots.

**Table 3:** Clinical events.

Outcome Variables	Syndrome Group			Loeys-Dietz Group by Causative Genes			
	Loeys-Dietz	Marfan	*p*	*TGFBR1*	*TGFBR2*	*SMAD3*	*p*
Number of individuals	83	83		26	40	17	
Deaths of any cause	8 (10%)	6 (7%)	0.781	2 (8%)	4 (10%)	2 (12%)	1.000
Deaths of any cause by age (years) ^1^	47 ± 22	50 ± 26	0.755	27–32	52 ± 20	26–81	0.570
Deaths by cause			0.867				0.829
Unknown	4 (50%)	2 (33%)		1 (50%)	2 (50%)	1 (50%)	
Aorta-related	3 (38%)	2 (33%)		1 (50%)	2 (50%)	0	
Heart-valve related heart failure	1 (13%)	1 (17%)		0	0	1 (50%)	
Heart failure of unknown cause	0	1 (17%)		0	0	0	
Proximal aortic surgery	33 (40%)	37 (45%)	0.637	12 (46%)	18 (45%)	3 (18%)	0.119
Proximal aortic surgery by age (years) ^1^	34 ± 14	35 ± 16	0.864	31 ± 10	36 ± 17	39 ± 7	0.512
Proximal aortic surgery by indication/location							
Urgent surgery (rupture/dissection)	11 (30%)	11 (30%)	0.800	5 (42%)	5 (28%)	1 (33%)	0.749
Surgery involving arch ^2^	2 (6%)	5 (14%)	0.434	2 (17%)	0	0	0.301
Proximal aortic surgery by technique			0.637				0.153
Aortic root reconstruction	23 (70%)	19 (51%)		8 (67%)	14 (78%)	1 (33%)	
Aortic root replacement (biological valve)	3 (9%)	3 (8%)		0	2 (11%)	1 (33%)	
Aortic root replacement (mechanical valve)	7 (21%)	15 (41%)		4 (33%)	2 (11%)	1 (33%)	
Distal aortic repair	8 (10%)	7 (8%)	1.000	1 (4%)	7 (18%)	0	0.085
Distal aortic repair by age (years) ^1^	41 ± 15	38 ± 11	0.536	23	43 ± 14		0.272
Distal aortic repair by indication/technique							
Urgent surgery (rupture/dissection)	2 (25%)	2 (29%)	1.000	0	2 (29%)	0	1.000
Elective surgery (true or false lumen expansion)	6 (75%)	5 (71%)		1 (100%)	5 (71%)	0	
Endovascular (versus surgical)	0	2 (29%)	0.200	0	0	0	
Distal aortic repair by location			0.322				0.500
Isolated thoracic aortic repair ^3^	4 (50%)	4 (57%)	0.793	0	4 (57%)	0	
Thoracoabdominal repair	1 (13%)	2 (29%)		0	1 (14%)	0	
Isolated abdominal aortic repair	3 (38%)	1 (14%)		1 (100%)	2 (29%)	0	
Mitral valve surgery	6 (7%)	14 (17%)	0.093	0	3 (8%)	3 (18%)	0.063
Mitral valve surgery by age (years) ^1^	47 ± 15	40 ± 14	0.239		21-50	50–65	0.077
Mitral valve surgery by indication							
Urgent surgery	0	1 (7%)	1.000	0	0	0	
Mitral valve surgery by technique			0.482				1.000
Reconstruction	3 (50%)	9 (64%)		0	1 (33%)	2 (67%)	
Biological valve prosthesis	1 (17%)	0		0	1 (33%)	0	
Mechanical valve prosthesis	2 (33%)	5 (36%)		0	1 (33%)	1 (33%)	
Number of events/individual			0.449				0.952
None	44 (53%)	41 (49%)		14 (54%)	20 (50%)	10 (59%)	
One	28 (34%)	24 (29%)		10 (39%)	12 (30%)	6 (35%)	
Two	7 (8%)	13 (16%)		1 (4%)	5 (13%)	1 (6%)	
Three	3 (4%)	5 (6%)		1 (4%)	2 (5%)	0	
Four	1 (1%)	0		0	1 (3%)	0	
First event in all individuals	39 (47%)	42 (51%)	0.756	12 (46%)	20 (50%)	7 (41%)	0.845
First event by age (years) ^1^	36 ± 14	36 ± 16	0.977	31 ± 10	37 ± 16	45 ± 13	0.141
First event by type			0.872				0.020
Death	2 (5%)	2 (5%)		0	1 (5%)	1 (14%)	
Proximal aortic surgery	31 (80%)	36 (86%)		12 (100%)	16 (80%)	3 (43%)	
Distal aortic intervention	2 (5%)	1 (2%)		0	2 (10%)	0	
Mitral valve surgery	4 (10%)	3 (7%)		0	1 (5%)	3 (43%)	
First procedure by	37	40		12	19	6	
Combined (versus isolated) procedure	0	4 (10%)	0.116	0	0	0	
Second event after non-lethal first event	11/37 (30%)	18/40 (45%)	0.239	2/12 (17%)	8/19 (42%)	1/6 (17%)	0.299
Second event by age (years) ^1^	46 ± 19	40 ± 15	0.387	23–32	47 ± 15	81	0.103
Second event by type			0.006				0.106
Death	3 (27%)	1 (6%)		1 (50%)	1 (13%)	1 (100%)	
Proximal aortic surgery	2 (18%)	1 (6%)		0	2 (25%)	0	
Distal aortic repair	6 (55%)	6 (33%)		1 (50%)	5 (63%)	0	
Mitral valve surgery	0	10 (56%)		0	0	0	
Distal aortic repair by age (years)	41 ± 15	38 ± 11	0.536	23	43 ± 14	41 ± 15	0.272
Distal aortic repair as second event by indication			0.318				1.000
True aneurysm	3 (50%)	0		1 (100%)	2 (40%)		
False lumen expansion after type A dissection	2 (33%)	4 (67%)		0	2 (40%)		
False lumen expansion of type-B dissection	1 (17%)	2 (33%)		0	1 (20%)		

^1^ With less than four individuals per variable, we present the range of age, or the age of a single individual. ^2^ Three of the five aortic arch procedures in Marfan syndrome (MFS) included a frozen elephant trunk procedure. ^3^ Two of four isolated thoracic aortic repair procedures in Marfan syndrome were carried out as a combined arch and descending aortic repair.

## Supplementary Materials

The following are available online at https://www.mdpi.com/2077-0383/8/12/2079/s1, Figure S1: ROC analysis of age as discriminator of risk for death; Figure S2: ROC analysis of age as discriminator of risk for proximal aortic surgery; Figure S3: ROC analysis of systemic score points as discriminator of risk for proximal aortic surgery; Figure S4: ROC analysis of aortic sinus diameters as discriminator of risk for proximal aortic surgery; Figure S5: Kaplan–Meier curve analysis of death and proximal aortic surgery according to indication for genetic testing; Figure S6: Kaplan–Meier curve analysis of distal aortic repair and mitral valve surgery according to indication for genetic testing; Table S1: Clinical manifestations according to indication for genetic testing in the Loeys-Dietz group; Table S2: Death of any cause in 83 individuals with Loeys-Dietz syndrome (LDS); Table S3: Proximal aortic surgery in 83 individuals with Loeys-Dietz syndrome (LDS); Table S4: Distal aortic repair in 83 individuals with Loeys-Dietz syndrome (LDS); Table S5: Mitral valve surgery in 83 individuals with Loeys-Dietz syndrome (LDS).
