# Supplementary material for: Case-matched Comparison of Cardiovascular Outcome in Loeys-Dietz Syndrome versus Marfan Syndrome
Source: J Clin Med. 2019 Nov 29;8(12):2079. doi: 10.3390/jcm8122079 (PMC6947024; doi:10.3390/jcm8122079)
Supplement: Supplementary file 1 [file jcm-08-02079-s001.pdf]

Supplementary Data

Figure S-1. ROC analysis of age as discriminator of risk for death

Receiver operating characteristic (ROC) curve analyzed the role of age at initial presentation as discriminator of risk for death of any cause, where the area under the curve was 0.609 (95% confidence interval .439 to .779; *P*=.177; left upper panel). Separate display of sensitivity and specificity identified 31.5 years of age as threshold of risk with a sensitivity and specificity of 50% (right upper panel). Kaplan–Meier curve analysis found a higher mean freedom from death at an age > 31.5 years (78±3 years, 95%CI 72–84) compared to an age ≤ 31.5 years (35±1 year, 95%CI 33–37; *P*<.001; lower panel).

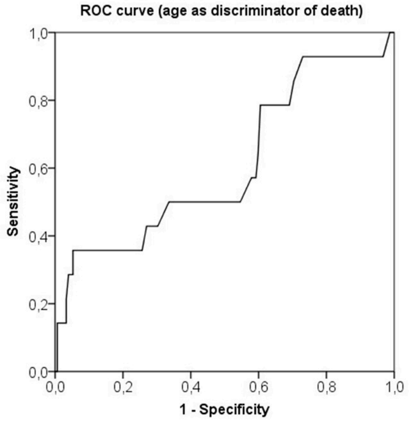

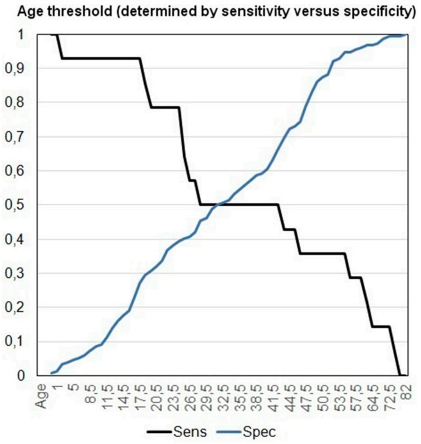

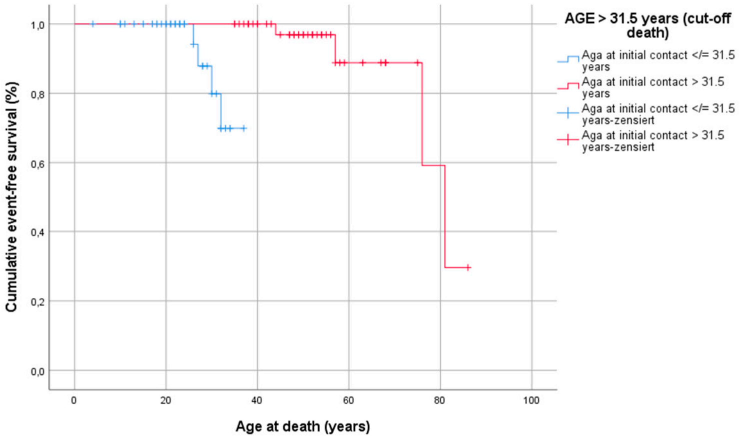

Log-rank test *P*<.001

Numbers at risk:

Age ≤ 31.5 years	40	28	0		
Age > 31.5 years	43	43	35	8	2

Figure S-2. ROC analysis of age as discriminator of risk for proximal aortic surgery

Receiver operating characteristic (ROC) curve analyzed the role of age at initial presentation as discriminator of risk for proximal aortic surgery, where the area under the curve was 0.646 (95% confidence interval .562- .730; *P*=.001; left upper panel). Separate display of sensitivity and specificity identified 33.5 years of age as threshold of risk with a sensitivity and specificity of 58% (right upper panel). Kaplan–Meier curve analysis found a lower mean freedom from proximal aortic surgery with earlier initial presentation to an expert center (age ≤ 33.5 years) than with presentation at an age beyond this threshold (28±1 years, 95%CI 26–31 versus 61±4 years, 95%CI 53–63; *P*<.001; lower panel).

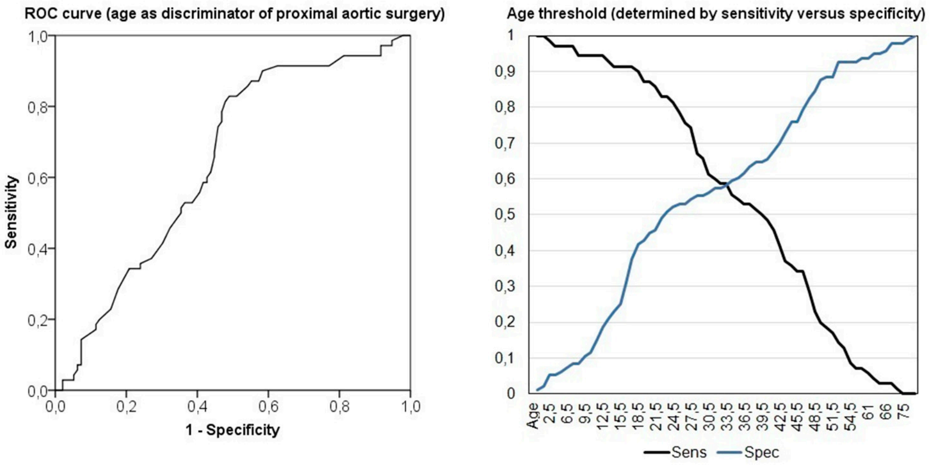

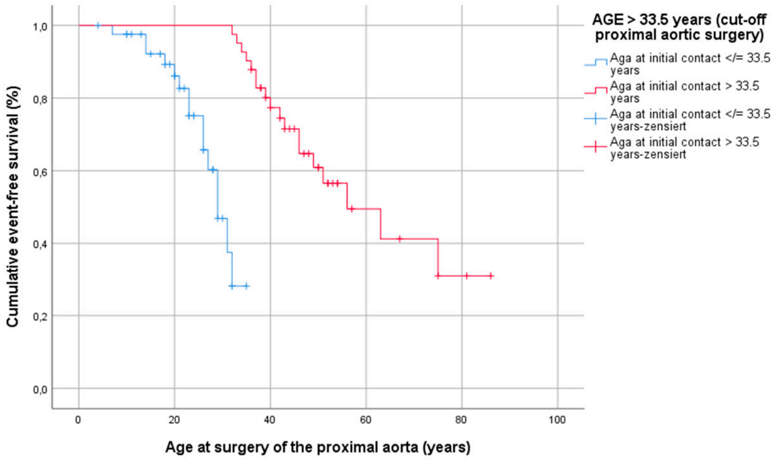

Log-rank test *P*<.001

Numbers at risk:

Age ≤ 33.5 years	42	27	0		
Age > 33.5 years	41	41	28	6	2

Figure S-3. ROC analysis of systemic score points as discriminator of risk for proximal aortic surgery

Receiver operating characteristic (ROC) curve analyzed the role of systemic score points as discriminator of risk for proximal aortic surgery, where the area under the curve was 0.621 (95% confidence interval .493 to .749; *P*=.068; left upper panel). Separate display of sensitivity and specificity identified 2.0 score points as threshold of risk with a sensitivity and specificity of 66% (right upper panel). Kaplan–Meier curve analysis found that a systemic score with > 2 points distinguished lower (45±4 year, 95%CI 36–53) from higher probability of freedom from proximal aortic surgery (55±5 years, 95%CI 46–64; *P*=.041; lower panel). In 4 individuals the systemic score was not assessed appropriately, and therefore these individuals were not included in the time to event analysis.

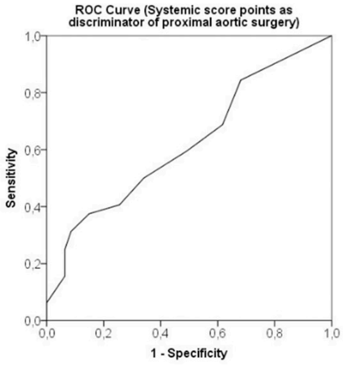

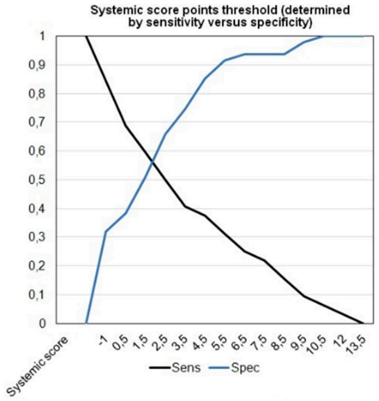

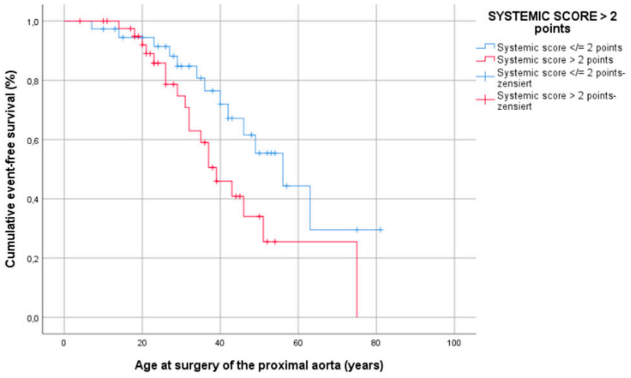

Log-rank test *P*=.041

Numbers at risk:

Systemic score ≤ 2 points	37	31	16	3	0
Systemic score > 2 points	42	32	9	1	0

Figure S-4. ROC analysis of aortic sinus diameters as discriminator of risk for proximal aortic surgery

Receiver operating characteristic (ROC) curve analyzed the role of aortic sinus diameters at initial presentation as discriminator of risk for proximal aortic surgery, where the area under the curve was 0.798 (95% confidence interval .674 to .923; *P*<.001; left upper panel). Separate display of sensitivity and specificity identified a sinus diameter of 3.45 cm as threshold of risk with a sensitivity and specificity of 69% (right upper panel). Kaplan–Meier curve analysis did not corroborate that an aortic sinus diameter ≤ 3.45 cm was a powerful discriminator of lower (64±6 years, 95%CI 53–76) from higher probability of freedom from proximal aortic surgery (50±4 years, 95%CI 42–58; *P*=.314; lower panel). For this time to event analysis we only considered individuals with native, non-operated aortic sinuses.

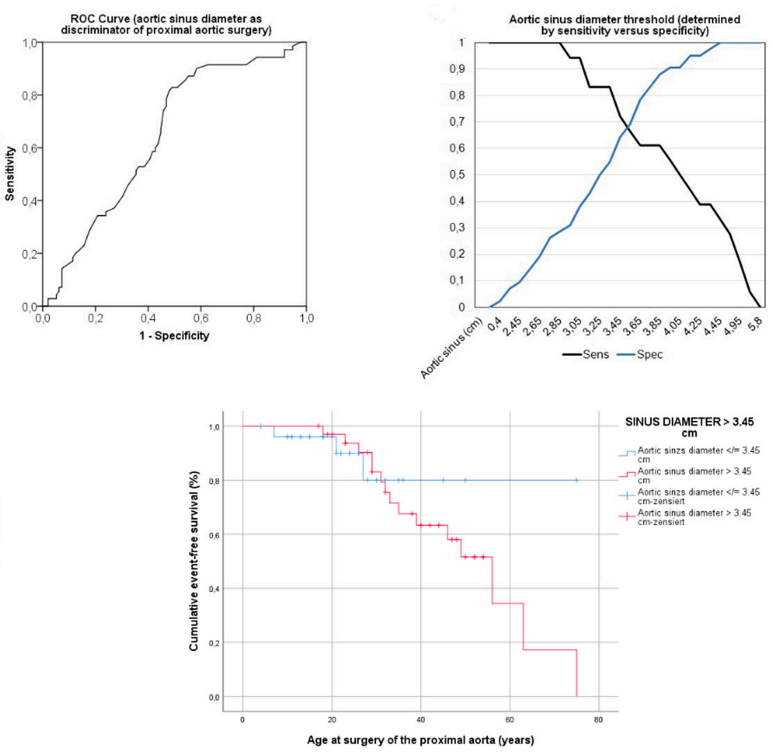

Log-rank test *P*=.314

Numbers at risk:

Aortic sinus diameter ≤ 3.45 cm	26	16	3	1	0
Aortic sinus diameter > 3.45 cm	34	30	14	2	0

Figure S-5. Kaplan–Meier curve analysis of death and proximal aortic surgery according to indication for genetic testing

Mean freedom from death (left panel) exhibited no inhomogeneity between the group with genetic testing performed for clinical suspicion of LDS (68±4 years; 95%CI 61–75) and the group with genetic testing performed as cascade screening in families with LDS (81±3 years, 95%CI 75–87; *P*=.091; left panel). In contrast, mean freedom from proximal aortic surgery was lower in group with genetic testing performed for clinical suspicion of LDS (43±3 years; 95%CI 37–48) than in the group with genetic testing

performed as cascade screening in families with LDS (70±5 years, 95%CI 60–80; *P*=.001; right panel).

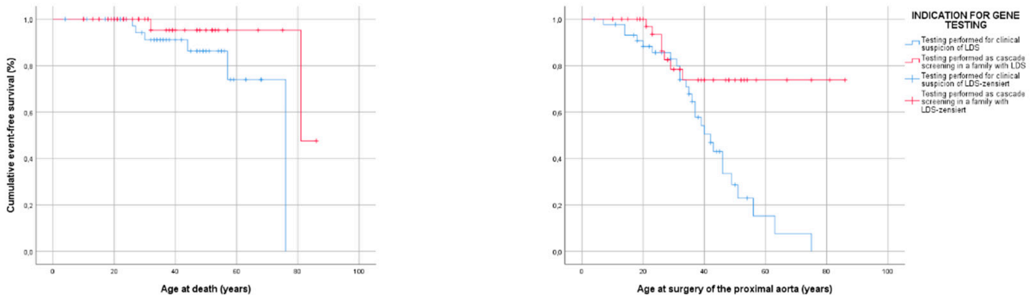

Log-rank test *P*=.091

Numbers at risk:					
Clinical suspicion of LDS	45	39	20	4	0
Cascade screening in LDS family	38	31	14	4	2

Log-rank test *P*=.001

Numbers at risk:					
Clinical suspicion of LDS	45	36	14	2	0
Cascade screening in LDS family	38	31	13	4	2

Figure S-6. Kaplan–Meier curve analysis of distal aortic repair and mitral valve surgery according to indication for genetic testing

Mean freedom from distal aortic repair exhibited no inhomogeneity between the group with genetic testing performed for clinical suspicion of LDS (67±3 years; 95%CI 60–73) and the group with genetic testing performed as cascade screening in families with LDS (75±4 years, 95%CI 68–82; *P*=.200; left panel). Mean freedom from mitral valve surgery also showed no inhomogeneity between the group with genetic testing performed for clinical suspicion of LDS (65±2 years; 95%CI 62–69) and the group with genetic testing performed as cascade screening in families with LDS (70±6 years, 95%CI 58–82; *P*=.302).

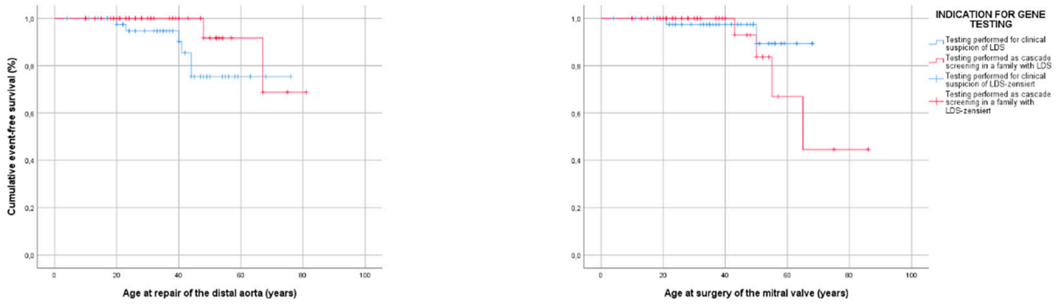

Log-rank test *P*=.200

Numbers at risk:

Clinical suspicion of LDS	45	38	20	4	0
Cascade screening in LDS family	38	31	14	4	1

Log-rank test *P*=.302

Numbers at risk:

Clinical suspicion of LDS	45	39	20	3	0
Cascade screening in LDS family	38	31	14	3	1

Table S-1. Clinical manifestations according to indication for genetic testing in the Loeys-Dietz group

Variable	Indication for genetic testing: Clinical suspicion of Loeys-Dietz syndrome	Indication for genetic testing: Cascade screening in families	*P*
Total number of individuals	45	38	
Age at initial contact (years)	35±17	34±20	.437
Age at final contact (years)	39±17	37±19	.459
Male sex	21 (48%)	22 (58%)	.380
Previous ischemic neurologic event	4/44 (9%)	2 (6%)	.685
Atrial septal defect	3 (7%)	1 (3%)	.621
Patent ductus arteriosus	5 (11%)	2 (5%)	.445
Bicuspid aortic valve	4 (9%)	1 (3%)	.369
Systemic score (points)	4.2±3.5	2.7±3.4	.032
Craniofacial severity index (points)	1.3±1.8	1.2±1.7	.782

LV ejection fraction (%)	60±12	64±10	.197
Indexed LVESD (mm/m2)	19±6	18±6	.264
Indexed LVEDD (mm/m2)	30±9	29±7	.368
Indexed left atrial diameter (mm/m2)	20±5	18±5	.177
Aortic sinus dimensions at initial presentation			
- Diameter (cm)[1]	3.7±.9	3.5±.6	.473
- Z-score[1]	2.6±4.1	1.7±1.9	.146
Aortic sinus dimensions at aortic surgery			
- Diameter (cm)	4.7±.6	5±.6	.405
- Z-score	4.4±2.5	6.6±2.3	.095
Moderate degree of MVR at baseline	2 (5%)	5 (15%)	.229
MV prolapse	15 (33%)	13 (34%)	1.000
MV leaflet prolapse location (N)	12	7	1.000

- Isolated anterior	6 (50%)	4 (57%)	
- Isolated posterior	1 (8%)	1 (14%)	
- Combined anterior and posterior	5 (42%)	2 (29%)	
Tricuspid valve prolapse	3 (7%)	2 (6%)	1.000

Table S-2. Death of any cause in 83 individuals with Loeys-Dietz syndrome (LDS)

Variable	Death of any cause		Univariate Cox regression analysis			*P*
	Absent (N = 75)	Present (N = 8)	Hazard ratio	Lower 95% CI	Upper 95% CI	
Age at initial contact (years)	33 ± 18	43 ± 22	.888	.823	.958	.002
Male sex	38 (51%)	5 (63%)	.681	.161	2.883	.602
Previous ischemic neurologic event	6/72 (8%)	0	.042	0	3228.209	.580
Systemic score (points)	3.35 ± 3.5	5.25 ± 3.4	1.158	.980	1.369	.086
Craniofacial severity index (points)	1.38 ± 1.8	.25 ± .71	.553	.231	1.322	.183

Left ventricular ejection fraction (%)	62 ± 11	56 ± 14	.947	.857	1.046	.283
Aortic sinus diameter (cm)	3.6 ± .9	3.5 ± .5	.446	.057	3.507	.443
Aortic sinus Z-score	2.2 ± 3.3	1.8 ± 2	1.831	.697	4.813	.220
Mitral valve prolapse	23 (31%)	5 (63%)	1.721	.343	8.638	.509
Tricuspid valve prolapse	4/72 (6%)	1/7 (14%)	3.895	.435	34.910	.224
Clinical suspicion of LDS for gene testing	40 (53%)	6 (75%)	1.96	.023	1.679	.137
SMAD3 (vs *TGFBR1*/*TGBR2*)[1]	15 (20%)	2 (25%)	1.020	.195	5.327	.981

CI identifies confidence interval; and N, numbers of events

[1]Variables were dichotomized for statistical reasons. The variables atrial septal defect or patent ductus arteriosus, or both, bicuspid aortic valve, and MV prolapse subtypes were not analysed for statistical reasons.

With only one variable yielding $P<.05$ on univariate analysis, we did not perform multivariable analysis.

Table S-3. Proximal aortic surgery in 83 individuals with Loeys-Dietz syndrome (LDS)

Variable	Proximal aortic surgery		Univariate Cox regression analysis			
	Not performed (N = 50)	Performed (N = 33)	Hazard ratio	Lower 95% CI	Upper 95% CI	*P*
Age at initial contact (years)	32 ± 20	37 ± 16	.895	.858	.933	<.001
Male sex	23 (46%)	20 (61%)	.681	.338	1.374	.283
Previous ischemic neurologic event	3/48 (6%)	3/32 (9%)	.777	.235	2.573	.680
Systemic score (points)	2.8 ± 2.9	4.6 ± 4.1	1.111	1.023	1.207	.012
Craniofacial severity index (points)	1.2 ± 1.74	1.36 ± 1.8	1.162	.955	1.413	.134
Left ventricular ejection fraction (%)	63 ± 10	60 ± 12	.986	.945	1.028	.502
Aortic sinus diameter (cm)	3.3 ± .6	4.3 ± .9	1.993	1.184	3.356	.009
Aortic sinus Z-score	1.7 ± 2	3.3 ± 4.9	1.212	1.047	1.404	.010
Mitral valve prolapse	18 (36%)	10 (30%)	.741	.347	1.582	.439

Tricuspid valve prolapse	2/46 (4%)	3 (9%)	2.908	.854	9.897	.088
Clinical suspicion of LDS for gene testing	20 (40%)	26 (79%)	.290	.124	.678	.004
SMAD3 (vs *TGFBR1*/*TGBR2*)	14 (28%)	3 (9%)	.323	.098	1.063	.063

	Multivariate Cox regression analysis			
Prediction of proximal aortic surgery	Hazard ratio	Lower 95% CI	Upper 95% CI	*P*
Age at initial contact (years)	.748	.658	.849	<.001
Systemic score (points)	1.175	.990	1.397	.065
Aortic sinus diameter (cm)[2]	4.176	1.721	10.133	.002
Clinical suspicion of LDS for gene testing	.544	.137	2.158	.387

CI identifies confidence interval; and N, numbers of events. The variables atrial septal defect or patent ductus arteriosus, or both, bicuspid aortic valve, and MV prolapse subtypes were not analysed for statistical reasons.

[1]Variables were dichotomized for statistical reasons.

[2]We only included aortic sinus diameters in multivariate analysis but not aortic sinus Z-scores, because both variables were not independent of each other.

Table S-4. Distal aortic repair in 83 individuals with Loeys-Dietz syndrome (LDS)

Variable	Repair of the distal aorta		Univariate Cox regression analysis			
	Not performed (N = 75)	Performed (N = 8)	Hazard ratio	Lower 95% CI	Upper 95% CI	*P*
Age at initial contact (years)	33±18	46±18	.954	.887	1.025	.197
Male sex	38 (51%)	5 (63%)	.669	.159	2.819	.583
Previous ischemic neurologic event	5/73 (7%)	1/7 (14%)	1.520	.177	13.078	.703
Systemic score (points)	3.3±3.4	6.3±4.6	1.215	1.021	1.445	.028
Craniofacial severity index (points)	1.19±1.7	2.0±2.5	1.407	.987	2.006	.059
Left ventricular ejection fraction (%)	62±11	64±10	1.030	.943	1.126	.509

Aortic sinus diameter (cm)	3.5±.8	5.2±.2	32.409	.590	1779.345	.089
Aortic sinus Z-score	2.4±2.6	-3.4±12	.852	.678	1.071	.171
Mitral valve prolapse	24 (32%)	4 (50%)	1.367	.322	5.805	.672
Tricuspid valve prolapse	3/71 (4%)	2 (25%)	6.818	1.305	35.610	.023
Clinical suspicion of LDS for gene testing	40 (53%)	6 (75%)	.363	.073	1.863	.227
SMAD3 (vs *TGFBR1*/*TGBR2*)	17 (23%)	0	.032	0	35.775	.337

	Multivariate Cox regression analysis[2]			
Prediction of distal aortic repair	Hazard ratio	Lower 95% CI	Upper 95% CI	*P*
Systemic score (points)	1.173	.953	1.445	.131
Tricuspid valve prolapse	1.895	.121	29.797	.649

CI identifies confidence interval; and N, numbers of events

[1]Variables were dichotomized for statistical reasons. The variables atrial septal defect or patent ductus arteriosus, or both, bicuspid aortic valve, and MV prolapse subtypes were not analysed for statistical reasons.

[2]We did not include the variables aortic sinus diameter and tricuspid valve prolapse in the multivariate model, because the 95%-CI in the univariate analysis of these variables were broad, and computation of a multivariate model with inclusion of these variables was not possible.

Table S-5. Mitral valve surgery in 83 individuals with Loeys-Dietz syndrome (LDS)

	Mitral valve surgery		Univariate Cox regression analysis			
Variable	Not performed (N = 77)	Performed (N = 6)	Hazard ratio	Lower 95% CI	Upper 95% CI	*P*
Age at initial contact (years)	32 ± 17	56 ± 20	1.003	.939	1.070	.937
Male sex	37 (48%)	6 (100%)	.014	0	10.656	.208
Previous ischemic neurologic event	6/75 (8%)	0	.038	0	5700.549	.591
Systemic score (points)	3.53 ± 3.5	3.75 ± 4.5	1.026	.816	1.291	.826
Craniofacial severity index (points)	1.29 ± 1.74	1 ± 2.24	1.045	.607	1.800	.873
Left ventricular ejection fraction (%)	62 ± 11	60 ± 8	1.006	.903	1.119	.919
Aortic sinus diameter (cm)	3.6 ± .8	4.4 ±1 .3	3.740	.445	31.456	.225
Aortic sinus Z-score	2.2 ± 3.2	-.46	.225	.005	10.150	.443
Mitral valve prolapse	22 (29%)	6 (100%)	96.626	.112	83025.628	.185

Tricuspid valve prolapse	4/76 (5%)	1/3 (33%)	7.762	.701	85.911	.095
Clinical suspicion of LDS for gene testing	44 (57%)	2 (33%)	2.404	.435	13.290	.315
SMAD3 (vs *TGFBR1*/*TGBR2*)[1]	14 (18%)	3/6 (50%)	4.400	.885	21.862	.070

CI identifies confidence interval; and N, numbers of events

[1]Variables were dichotomized for statistical reasons. The variables atrial septal defect or patent ductus arteriosus, or both, bicuspid aortic valve, and MV prolapse subtypes were not analysed for statistical reasons.

Since no variable yielded $P<.05$ on univariate analysis, we did not perform multivariable analysis.
